# Iatrogenic Foot Drop After Anterior Cruciate Ligament Reconstruction With Peroneus Longus Tendon Autograft: Report of a Rare Case

**DOI:** 10.7759/cureus.26476

**Published:** 2022-06-30

**Authors:** Umesh Yadav, Mudit Nemani, Ashish Devgun, Manmeet Malik, Gaurav K Agrawal

**Affiliations:** 1 Orthopaedics, Pandit Bhagwat Dayal Sharma Post Graduate Institute of Medical Sciences, Rohtak, IND; 2 Orthopaedics and Trauma, Pandit Bhagwat Dayal Sharma Post Graduate Institute of Medical Sciences, Rohtak, IND; 3 Orthopaedics, Post Graduate Institute of Medical Sciences, Pandit Bhagwat Dayal Sharma University of Health Sciences, Rohtak, IND

**Keywords:** arthroscopic acl reconstruction, common peroneal neuropathy, foot drop, peroneus longus tendon, anterior cruciate ligament (acl) reconstruction

## Abstract

Reconstruction of the anterior cruciate ligament using autografts is a common procedure performed in the modern era. The peroneus longus tendon is an upcoming graft with several advantages over traditional autografts. It has minimal donor site morbidity in relation to biomechanical properties of the ankle. Common peroneal nerve injury during harvest is a theoretical concern while harvesting the peroneus longus tendon. The following case highlights the importance of careful surgical technique and timely intervention while dealing with such complications. A 25-year-old male suffered an anterior cruciate ligament rupture while wrestling. He had an unstable knee and difficulty performing daily activities. He underwent an arthroscopic anterior cruciate ligament reconstruction using peroneus longus tendon autograft. Following surgery, the patient reported a foot drop and decreased sensations over the dorsum of the foot. The patient was advised of a foot drop splint and neuroprotective medications. Neurophysiological studies were not performed since they cannot differentiate between partial and complete nerve injury in the first week after injury. A surgical exploration of the nerve was done. An intraneural hematoma was found with contusions over the peroneus longus tendon. Neurolysis was performed to decompress the nerve. The functioning of the anterior cruciate ligament was satisfactory during follow-up. An advancing Tinel’s sign was noted on follow-up. The patient finally recovered after a 3-month follow-up.

## Introduction

An anterior cruciate ligament (ACL) rupture is a common injury among athletes. The two major types of grafts used in ACL reconstruction are autografts and allografts. Some of the advantages of an allograft are minimal morbidity to the donor site, decreased surgical and rehabilitation times, less pain, and better cosmesis. Peroneus longus tendon (PLT) is an emerging option with minimal donor site morbidity and favorable biomechanical properties [1].

Most popular autografts are harvested from the knee which carries many disadvantages, such as resulting in decreased strength of hamstrings and quadriceps-hamstring imbalance after harvest. Peroneus longus tendon is an upcoming graft in ACL reconstruction. It demonstrated minimal post-operative morbidity from the ankle donor site because of biomechanical inconvenience [2].

Up to 88% risk of damage to the saphenous nerve has been reported while harvesting hamstring tendon (HT) autograft [3], which is the principal complication. This can be reduced to 14.9% by changing the harvesting approach [4]. Reports of such injuries following PLT graft harvest are few. Cadaveric studies show a theoretical risk of the common peroneal nerve and sural nerve injuries following the harvest of PLT autograft [5].

We hereby report a case of peroneal nerve injury during harvesting the peroneus graft leading to foot drop in the post-operative period. Nerve exploration was performed and contused muscle fibers along with hematoma in the nerve were diagnosed which was treated by neurolysis.

## Case presentation

A 25-year-old wrestler presented to us in the Out-patient Department with complaints of knee instability and pain during everyday activities such as climbing or coming down stairs, squatting, giving out of the knee during walking, and inability to stand on the affected limb for prolonged periods. He had a history of a twisting injury to the knee while wrestling 2 months ago. Physical examination showed a positive Lachman's test, pivot shift test, and anterior drawer test. Radiographs showed no bony injury to the knee. MRI revealed a complete tear of the ACL. The patient was taken up for arthroscopic ACL reconstruction using PLT autograft after completing the necessary pre-anesthetic workup.

The PLT graft was harvested by making a 2-cm longitudinal incision over the posterior border of the ipsilateral lateral malleolus. The superior peroneal retinaculum was explored and the peroneus longus tendon was isolated. The distal part of the peroneus longus tendon was cut and sutured. The proximal end was released by the tendon stripper. The length and diameter of the tendon were measured after separating it from muscle tissue. The PLT graft was folded through the middle and a double-loop graft was obtained which was 14 cm in length and 8.0 mm in diameter. The ACL remnant was visualized arthroscopically and removed. After creating proper femoral and tibial tunnels, the double-loop PLT graft was inserted and fixed using an endobutton on the femoral side. An interference screw was used to fix the graft on the tibial side.

Immediately after recovery from spinal anaesthesia, the patient reported a foot drop and decreased sensations over the dorsum of the foot. The patient was advised of a foot drop splint and neuroprotective medications. Surgical exploration of the nerve was done to rule out transection of the nerve while using the tendon stripper. An intraneural hematoma was found with contusions over the peroneus longus tendon. Decompression of the nerve was done by neurolysis. During follow-up, the ACL function was found to be satisfactory. An advancing Tinel's sign was noted on follow-up. The patient finally recovered after a 3-month follow-up.

**Figure 1:**
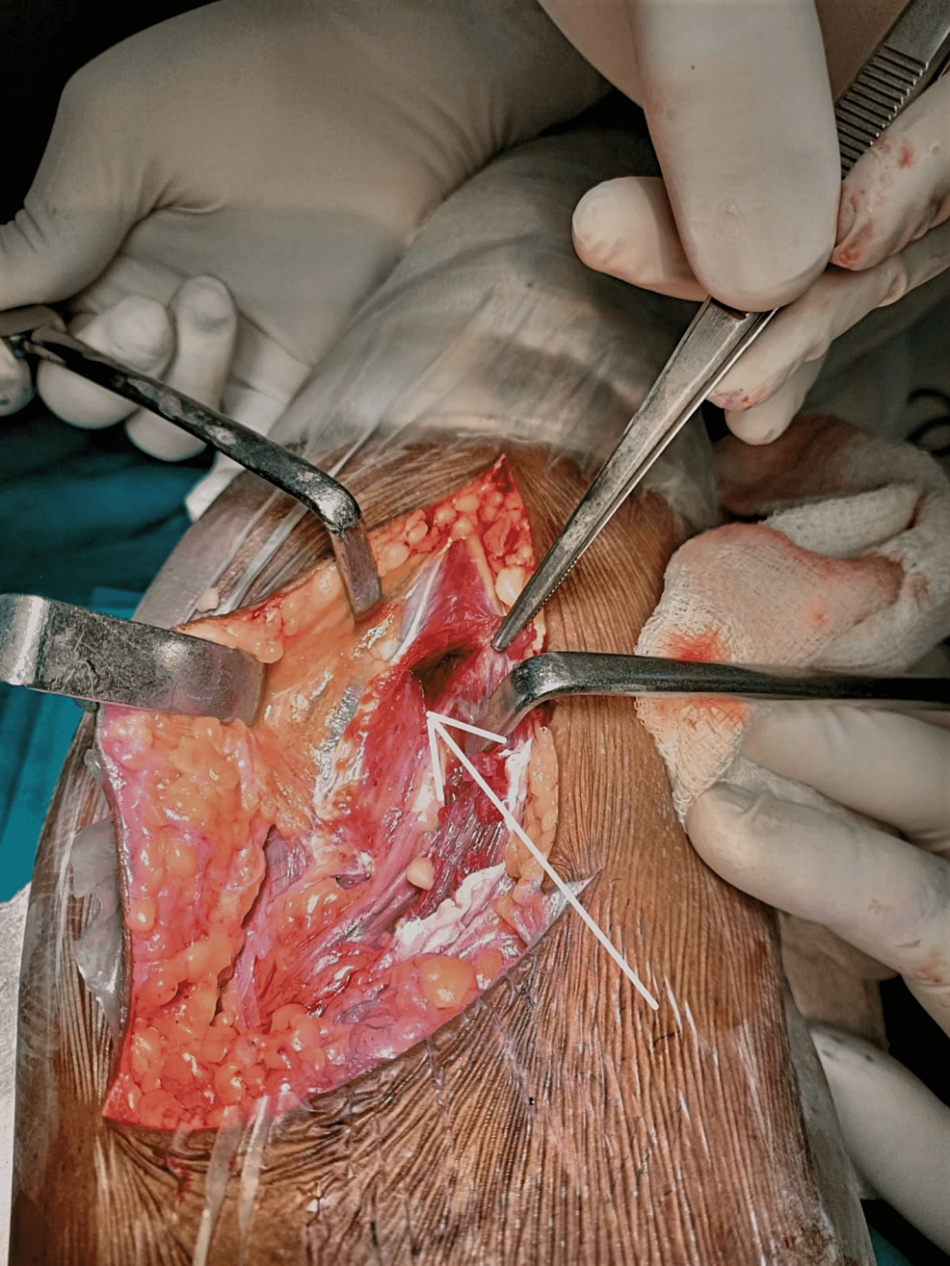
Contusion in the peroneus longus (white arrow)

**Figure 2:**
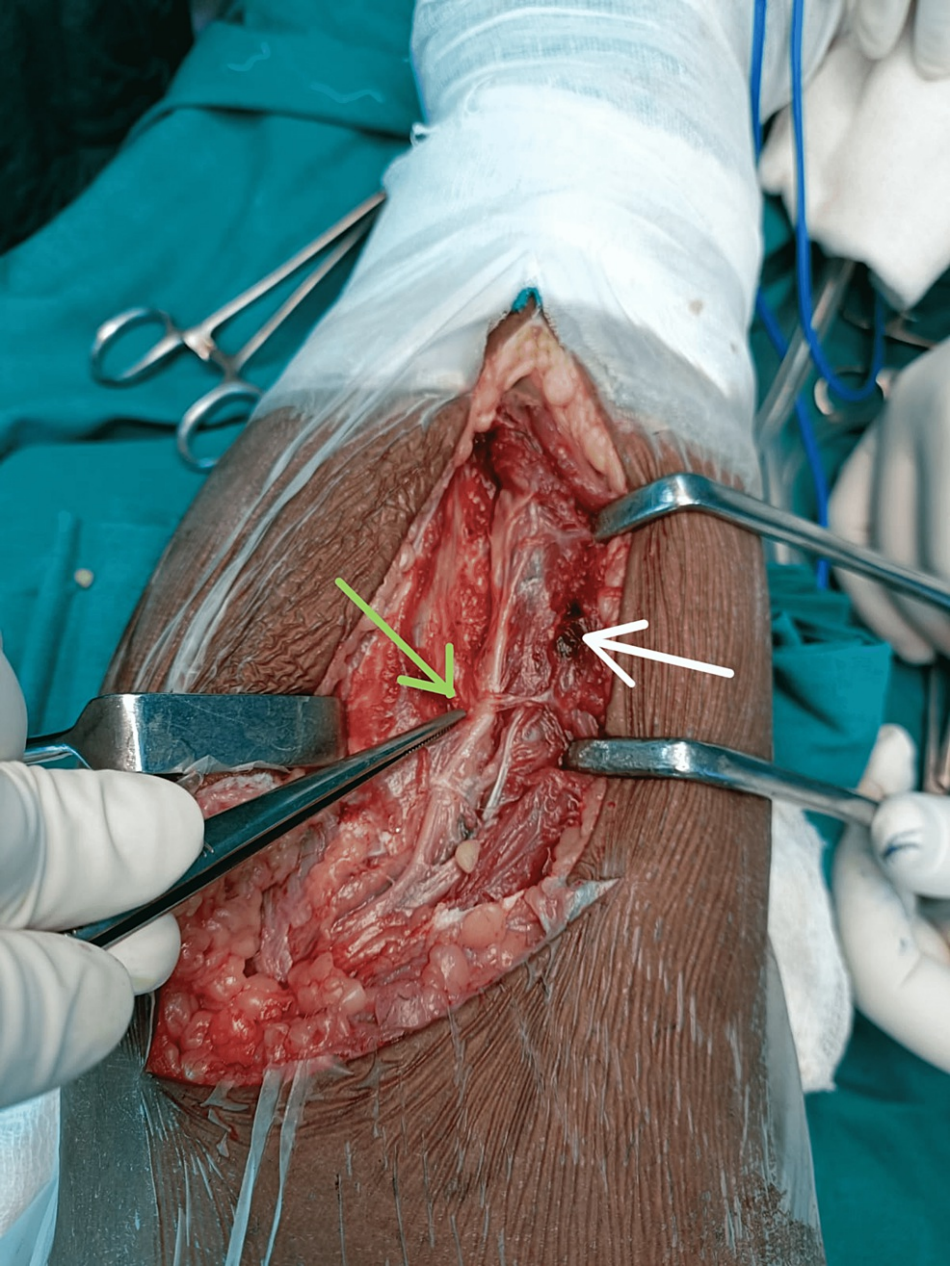
Hematoma at the level of bifurcation of common peroneal nerve Green arrow - intraneural hematoma at the level of bifurcation of common peroneal nerve; white arrow - contusion in the peroneus longus muscle

Figure 1 and Figure 2 show the contusion in the peroneus muscles and the intraneural hematoma during surgical exploration, respectively.

## Discussion

Peroneus longus autografts are considered a safe and effective alternative for HT autografts due to functionally acceptable biomechanical results and reasonably low donor-site morbidity. However, graft harvesting may be associated with some rare complications like peroneal nerve injury as reported in the present case.

Harvesting tendon autografts using a tendon stripper is associated with a low to moderate risk of nerve injury as has been demonstrated in previous studies. Injury to the infrapatellar branch of the saphenous nerve (IPBSN) was seen mainly while harvesting hamstring tendons using vertical incisions. It was seen that they were associated with a minor possibility of complete recovery within the first year after surgery [6]. Vertical incisions have the highest incidence of injury to the infrapatellar branch of the saphenous nerve (IPBSN), with persistent hypoesthesia, the largest area of sensory loss, and the poorest overall subjective outcome. Oblique and transverse incisions had similar results. Injury to the sartorial branch of the saphenous nerve (SBSN) was found to be equally common in all three incisions used. However, normal daily activities in the patients were not impaired by the said injury. An oblique incision was recommended by Sabat and Kumar for harvesting hamstring grafts as they were found to be safer [7].

The tendon stripper lies near the peroneal and sural nerves when the peroneus longus tendon (PLT) is harvested as demonstrated by He et al. The distance from the sural nerve to the PLT was found to increase from 0 to 2 cm proximal to the lateral malleolus (LM). The distance between the tendon stripper and the deep peroneal nerve was approximately 52 mm. The average distance to the PLT branch of the peroneal nerve was approximately 29 mm. The superficial peroneal nerve was approximately 5.2 mm away from the end of the stripper and ran parallel to it. He did not find transection injuries in the 10 legs harvested [8]. Nerve injuries following tendon harvest using tendon strippers have been reported in HT and patellar tendon autografts. The saphenous nerve is most commonly injured in such cases. Ruffilli et al described the anatomical considerations during harvesting of HT pointing to the course of the saphenous nerve and suggested an oblique incision to reduce the risk of injury to the saphenous nerve [9]. Mens et al. described a case of neuropraxia of the common peroneal nerve (CPN) during harvesting of HT grafts for ACL reconstruction; the patient, in that case, recovered after neurolysis [10].

Blakey and Biant published a case report where the CPN had been transected during harvesting of the semitendinosus and gracilis tendons for the reconstruction of the ACL. Immediately postoperatively the patient had a painless CPN palsy. A complete lesion of the CPN, with loss of conduction and fibrillation potentials, was found in the tested muscles two months after surgery. On surgical exploration, they found a complete transection of the CPN about 20 cm proximal to the tip of the fibula. Sural nerve grafts were utilized to reconstruct the common peroneal nerve. At 15 months of follow-up, they found the incomplete recovery of dorsiflexion and eversion of the foot with incomplete sensory recovery [11]. 

Another case reported transient superficial peroneal nerve palsy after ACL reconstruction. The patient complained of numbness and tingling in the area innervated by the superficial peroneal nerve. Physiotherapy was started 8 hours post-operatively. The patient regained dorsiflexion and eversion the subsequent day, and there was a complete recovery of function after two days [12].

Theoretical risk of injury to CPN and the sural nerve exists while harvesting PLT autografts near the tendon. To our knowledge, this is the first reported case of CPN injury while harvesting the PLT. Table 1 shows the nerve injuries that have been reported in various studies.

**Table 1:** Nerve injuries reported while using various grafts

Study (year)	Graft	Nerve injury reported
Haviv et al (2017) [6]	Hamstring	Infrapatellar branch of the saphenous nerve (IPBSN)
Sabat et al (2013) [7]	Hamstring	Infrapatellar branch of the saphenous nerve (IPBSN) during vertical incisions Sartorial branch of the saphenous nerve (SBSN
Ruffilli et al (2017) [9]	Hamstring	Saphenous nerve
Mens et al (2021) [10]	Hamstring	Common peroneal nerve (CPN)
Blakey et al (2008) [11]	Semitendinosus and gracilis	Common peroneal nerve (CPN)

Table 2 summarizes some of the surgical tips and tricks to avoid nerve injury. 

**Table 2:** Tips and tricks to avoid nerve injury during harvesting of grafts

Serial No.	Methods to avoid nerve injury
1.	While harvesting the graft, about 5 cm of the proximal end should be left untouched to protect the common peroneal nerve (CPN) from injury.
2.	While positioning the tendon stripper, the distal end of the tendon should be properly visualized and the surgeon should have knowledge of the direction of the fibers.
3.	Application of appropriate tension at the tendon and a controlled motion of the harvesting device would lessen the risk of iatrogenic injury.
4.	Blunt-tipped instruments may lessen the risk of such complications.

The nerves are definitely at risk during the harvesting of tendons using tendon strippers. Bonney stated that the nerve should be assumed to be cut if a complete paralysis is seen in the region of distribution of the nerve post-operatively and the incision used lies over the main nerve. Prompt exploration and repair of the nerve should be done in such cases [13]. Exploration of the nerve should also be done even when a scar cannot be seen, and a blind operation has been performed over the course of the nerve. The present nerve injury can be attributed to the over-enthusiasm of the resident in harvesting the graft and poking the nerve with the tendon stripper too proximal.

## Conclusions

PLT autografts are useful alternatives in arthroscopic ACL reconstruction with good mechanical properties, minimal donor site morbidity, and minimal risk of complications. However, injury to the CPN can happen during the graft harvesting proximally, which can be avoided by increasing vigilance during tendon harvest. Timely intervention can prevent permanent damage to the nerve structure.
